# Reproductive performance and physiological responses of Barki does fed *Spanish panicum* and cassava (*Manihot esculenta*) as alternatives to berseem hay

**DOI:** 10.1186/s12917-025-05267-w

**Published:** 2026-02-02

**Authors:** Emad F. EL-Maghraby, Ahmed S. El-Hawy, Ferial M. Sahwan, Muhammed Ahmed-Hilmy El-Rayes, Mona M. Elghareeb, Afaf H. Zedan, Gehad E. Elshopakey, Hisham A. Abdelrahman, Shimaa A. Sakr

**Affiliations:** 1 Department of Buffalo Breeding Research, Animal Production Research Institute, Agricultural Research Center, Dokki, Giza, Egypt; 2 Animal Production Department, Faculty of Agriculture, Al-Azhar University, Cairo, Egypt; 3 Animal and Poultry Production Division, Desert Research Center, Cairo, Egypt; 4 Animal Breeding and Production, Animal Husbandry and Animal Wealth Development Department, Faculty of Veterinary Medicine, Alexandria University, P.O. Box 22758, Edfina, Egypt; 5 Department of Animal Physiology, Animal and Poultry Production Division, Desert Research Center (DRC), Cairo, Egypt; 6 Department of Physiology, Faculty of Veterinary Medicine, Mansoura University, Mansoura, Egypt; 7 Animal Production Research Institute, Agriculture Research Center, Dokki, Giza, Egypt; 8 Department of Clinical Pathology, Faculty of Veterinary Medicine, Mansoura University, Mansoura, 35516 Egypt; 9 Department of Biology, Marine Biology and Environmental Science, Roger Williams University, Bristol, Rhode Island 02809 USA; 10 Department of Animal Wealth Development, Faculty of Veterinary Medicine, Mansoura University, Mansoura, Egypt

**Keywords:** Barki goats, Panicum, Cassava leaf meal, Berseem hay, Reproductive performance, Immunity

## Abstract

**Problem/Gaps:**

Small ruminants in developing countries often rely on Berseem hay (BH) as a primary fodder, but alternative, locally available, and nutritious feed sources are needed to improve reproductive performance and health.

**Objectives:**

This study investigated the use of *Spanish Panicum* and cassava (*Manihot esculenta*) leaf meals as partial replacements for BH in the diets of Barki doe goats during the spring and autumn breeding seasons.

**Methods:**

A total of 42 healthy does (2–3 years old) were divided into three dietary groups: G1 (control, 40% concentrates + 60% BH), G2 (BH replaced by *Panicum*), and G3 (BH replaced by cassava). After a 4-week diet adaptation period, goats were fed their respective rations throughout mating until weaning. Blood samples were collected biweekly from the jugular vein of all does. Complete blood counts were measured immediately, and plasma was separated and stored for subsequent biochemical and immunological analyses.

**Results:**

Both *Panicum* and cassava leaf meals significantly improved reproductive performance compared to BH. Conception and kidding rates reached 100% in spring, and the *Panicum* group achieved the highest fecundity (220%) and survival rate (95.5%) in autumn. Hematological parameters, including total erythrocyte count, hemoglobin, PCV, MCHC, and leukocyte count, increased significantly in the cassava group (*P* < 0.0001). Pregnant does fed cassava also showed elevated plasma total protein, albumin, globulin, glucose, and ATP levels (*P* < 0.0001). Immune status improved in both *Panicum* and cassava groups, with higher plasma TAC, IgG, and complement proteins C3 and C4 (*P* < 0.0001).

**Conclusion:**

Our findings indicate that incorporating *Panicum* and cassava leaf meals as alternatives to Berseem hay enhances reproductive efficiency, hematological health, and immunity in Barki does during breeding seasons, supporting their use as effective local feed resources.

## Introduction

Goats are a vital component of smallholder farming systems, contributing significantly to rural livelihoods by providing meat, milk, skin, fiber, and manure [1]. Compared with large ruminants, goats possess superior adaptability to harsh environmental conditions and low-quality feed resources, making them particularly valuable in arid and semi-arid regions [2]. Despite their adaptability, goat production in Egypt remains constrained by seasonal fluctuations in forage availability and quality, alongside increasing feed costs [3]. These challenges were most critical during the dry season, when poor-quality herbage fails to meet the nutritional demands of breeding and pregnant does, leading to weight loss, suppressed fertility, and reduced productivity [2]. Consequently, the search for alternative, cost-effective, and nutritionally adequate feed resources has become essential to sustain goat performance throughout the year.

Among various forage alternatives, *Spanish Panicum* (Guinea grass) and cassava leaves have gained attention due to their favorable agronomic traits and nutritional composition [4]. *Spanish Panicum* (*Panicum maximum*), a perennial grass of the *Poaceae* family, was widely distributed in tropical and subtropical regions of Africa and valued for its high biomass yield, regenerative capacity, and tolerance to salinity in both soil and water [5, 6]. It responds efficiently to nitrogen fertilization, remains palatable at all growth stages, and provides substantial forage even under marginal conditions, making it one of the most productive tropical grasses [7]. Nutritionally, *Panicum maximum* contains approximately 10–18% crude protein, 60–65% total digestible nutrients (TDN), and 25–35% crude fiber, depending on the growth stage and fertilization regime, indicating its potential as a balanced roughage source for ruminants [8].

Similarly, cassava (*Manihot esculenta*) is a major tropical crop whose leaves and by-products serve as valuable livestock feed [9]. Cassava leaves are rich in nutrients, with crude protein content ranging between 16% and 40% of dry matter, and are abundant in essential vitamins (B₁, B₂, and C), minerals, and carotenoids [10, 11]. Early-harvested cassava foliage (around three months of growth) exhibits lower tannin content and higher protein concentration, enhancing its digestibility and nutritive value [12]. Although cassava leaf protein is slightly deficient in methionine and lysine, its amino acid profile remains comparable to that of high-quality protein sources such as soybean or fish meal [13]. Moreover, incorporating cassava leaf meal in small ruminant diets has been reported to improve feed efficiency, growth rate, and gastrointestinal development under resource-limited conditions [14, 15].

A comparative summary of these forages shows that Bersem hay typically contains about 14–20% crude protein and 55–60% TDN, comparable to *Panicum* and cassava leaf meal in nutrient density, but its availability is restricted to specific growing seasons [16]. In contrast, *Panicum* and cassava can be cultivated or harvested throughout much of the year, offering practical and economic advantages in feed management [17].

Despite these benefits, limited information is available on the potential of replacing Berseem hay (BH) with Spanish Panicum or cassava leaf meal in goat diets, particularly for indigenous breeds such as *Barki* goats under Egyptian conditions and during different breeding seasons. Addressing this knowledge gap is critical for improving reproductive performance, physiological resilience, and immune competence under feed-limited environments. Therefore, this study aimed to evaluate the effects of replacing Berseem hay with Spanish Panicum or cassava leaf meals in the diets of Barki does during the spring and autumn breeding seasons on their reproductive performance, physiological responses, and immune status. We hypothesized that *Panicum* and cassava leaf meals, due to their high protein content, favorable nutrient profiles, and bioactive compounds, would sustain or enhance reproductive efficiency, physiological balance, and immune function compared with Berseem hay, while reducing feed costs.

## Materials and methods

The study was conducted on a private farm located in the northwestern coastal region of Egypt, near the Mediterranean Sea, west of Alexandria. The geographical coordinates of the study area are approximately 31°N latitude and 29°E longitude.

### Experimental design and diets

Forty-two healthy Barki does Aged 2–3 years old with an average body weight of 19–31 kg were used. All animals used in this experiment were injected with (Estrumate 1 ml/head contained 250 µg/ml injectable solution Cloprostenol, manufactured by Vet Pharma Friesoythe GmbH, Germany, Reg No:1887) to induce estrous synchronization and (Recepteen 1.5 ml/head contained 40 µg/ml injectable solution GnRH, manufactured by ADWIA Vit Bureau 175, 90 South Road 2nd Sector, 5th Settlement, New Cairo, Cairo, Egypt). Half of the goats (21 does) entered the mating season in March 2020 and gave birth in August 2020, while the other half (21 does) entered the mating season in September 2020 and kidded in February 2021. The experimental animals were kept in a semi-open shaded yard and kept under the same managerial conditions during the experimental periods. The experimental periods of each half contained three intervals: mating, pregnancy, and suckling.

Animals in each interval were divided into three equal groups: The first group received the control diet consisting of 40% concentrates and mixture (CFM), plus 60% berseem hay (BH), while the second and third groups had BH replaced by edible parts of *Spanish Panicum* and Cassava *Manihot esculenta*, respectively. The formulated diet is shown in Table 1.

**Table 1:** Formulated diet and chemical composition of the feedstuffs, concentrate feed mixtures, and experimental diets

Ingredient	Treatments		
	Control	Panicum	Cassava
Berseem hay	60	0	0
Spanish Panicum	0	60	0
Cassava	0	0	60
Yellow corn	17.2	17.2	17.2
Un-decorticated cotton meal	10	10	10
Wheat bran	10	10	10
Molasses	1.4	1.4	1.4
Limestone	0.8	0.8	0.8
Common salts	0.4	0.4	0.4
Minerals mixture	0.2	0.2	0.2
Total	100	100	100
Chemical composition (on DM % basis)			
Dry matter	93.63	81.30	63.19
Organic matter	90.85	89.35	90.06
Crude protein	12.32	16.07	19.70
Crude fiber	28.51	19.96	22.22
Ash	8.94	10.44	9.73
Ether extract	1.96	2.74	3.09
Nitrogen-Free Extract 48.26		50.77	45.24

The CFM consisted of 43% (17.2) yellow corn, 25% (10) undecorticated cotton meal, 25% wheat bran (10), 3.5% (1.4) molasses, 2% (0.8) limestone, 1% (0.4) common salts and 0.5% (0.2) minerals mixture

Animals were fed diets to cover their nutrient allowances corresponding to the physiological and productive stage according to [18]. Does were adapted to their diets for 4 weeks as a preliminary period, and then fed experimental rations before mating season and continued to wean their kids (suckling period, 12 weeks). All animals were fed twice daily (at 9 a.m. and 4 p.m.) while fresh water was available at all times. The chemical composition of DM% (DM, OM, CP, EE, NFE, and Ash) of feedstuffs was analyzed according to [19]. The animals were weekly weighed with an electronic balance for small ruminants to calculate the weight gain/ animal/ week, along with adjusting the concentrated forage quantities provided to each group in light of the total live body weight/ group.

### Reproductive performance

Reproductive performance of Barki does was evaluated using the following parameters:

Conception rate (%) = (Number of pregnant does / Number of mated does) × 100.

Kidding rate (%) = (Number of does giving a live kid / Number of mated does) × 100.

Fecundity (%) = (Number of kids born / Total number of does exposed to bucks) × 100.

Survival rate in kids (%) = (Number of kids weaned / Number of kids born) × 100.

Litter size (prolificacy) = Number of kids born / Number of does kidding.

Gestation period (days) = Number of days from mating until kidding.

These calculations were applied consistently for all experimental groups to evaluate the effect of dietary treatments on reproductive performance.

### Sample collection

Blood samples (*n* = 10/ group) were collected biweekly from the jugular vein into clean test tubes with anticoagulant (EDTA). Blood samples were divided into two parts. The complete blood count (CBC) was applied in the first part. The second part was centrifuged at 3000 rpm for 20 min to obtain plasma. The resulting plasma was preserved at − 80 °C for subsequent analyses of biochemical, inflammatory, and antioxidant indicators.

### Blood cell count

Complete blood components (CBC), including count of red (RBCs, x10^6^/mm^3^) and white blood cells (WBCs, x10^3^/mm^3^), hematocrit value (HCT, %), hemoglobin (Hb, g/dl) concentration, and differential leukocytic count in the whole blood were immediately measured after collection, according to the methods of Barger [20].

### Biochemical analysis

The plasma concentrations of total proteins (MD1001291), albumin (MX1001020), total lipids (Ref: 1001270), triglycerides (MX41031), total cholesterol (1001090), glucose (MD41011), AST (MD41264), ALT (SP41274), GGT (MX41288), ALP (MD41233), creatinine (MD1001111), BUN (Ref. TK41041) and IgG (MD1103004) were assayed using kits purchased from Spinreact company (Girona, Spain). Total antioxidant capacity (TAC) was evaluated spectrophotometrically using commercial reagent kits (Bio-diagnostic Co., Cairo, Egypt). However, globulin concentration was calculated as the difference between total protein and albumin, and the albumin-to-globulin (A/G) ratio was derived by dividing albumin by globulin, as described by Kaneko [21]. Additionally, adenosine triphosphate (ATP, MBS2000060), complement components C3 (MBS030286) and C4 (MBS743750), and pro-inflammatory cytokines including interleukin-1 (IL-1, MBS705809), interleukin-2 (IL-2, MBS4500333), interleukin-6 (IL-6, MBS025544), and tumor necrosis factor-alpha (TNF-α, MBS263127) were quantified from undiluted plasma samples using enzyme-linked immunosorbent assay (ELISA) kits (MyBioSource, Inc., San Diego, CA, USA). Thyroid hormones triiodothyronine (T3, ab108685) and thyroxine (T4, ab178664) were measured using ELISA kits supplied by Abcam (Cambridge, UK).

### Statistical analysis

Data were expressed as LSM ± SE. Data on the litter size, gestation period, and blood hematological, biochemical, and immunological parameters were statistically analyzed by General Linear Model (GLM) using procedures of SAS [22]. The model includes the effect of treatments (three variables), the breeding seasons (spring and autumn), and their interaction. Means were compared using the LSMEANS/ PDIFF of the same procedure. Differences were considered significant at *P* ≤ 0.05. The Chi-square (χ2) test was used to analyze the conception rate, kidding rate, fecundity, and survival rate of kids using the procedures of SAS [22].

## Results

### Economic evaluation of different forage types

The economic analysis of the feeding trials revealed noticeable differences among the tested forages in terms of feed cost and production efficiency. The total feed cost per doe was lowest in animals fed Panicum, followed by those fed cassava, whereas the Berseem hay (BH) group recorded the highest feed cost. When the economic efficiency was calculated based on the ratio between the value of produced kids and the total feed cost, both Panicum and Cassava groups showed higher net returns and superior economic efficiency compared with the BH group. The improvement in profitability could be attributed to the lower price of Panicum and cassava forages and their positive effects on reproductive performance, fecundity, and kid survival rate.

These findings indicate that *Panicum* and cassava can be used as cost-effective alternatives to BH, contributing to improved production efficiency and sustainable livestock management under limited forage availability.

### Reproductive performance

The present results demonstrated that there were no significant differences among the experimental groups in the gestation length. Additionally, Barki does feed various forages in the autumn season had an overall mean gestation length similar to those fed *Panicum*, cassava, or Berseem hay during spring (Table 2).

**Table 2:** Effect of feeding different types of forages on the gestation length and litter size (LSM ± SE) of Barki does

Item	Season	Experimental groups			Overall	*P*-value		
		Berseem hay	Panicum	Cassava		Season	Group	Interaction
Gestation length (d)	Autumn Spring Overall	150.0 ± 1.05 150.9 ± 1.05 150.4 ± 0.74	151.4 ± 0.99 152.5 ± 0.99 151.9 ± 0.70	151.8 ± 1.05 152.4 ± 0.99 152.1 ± 0.72	151.1 ± 0.59 151.9 ± 0.58	0.301	0.223	0.972
Litter size	Autumn Spring Overall	1.4 ± 0.31^b^ 1.2 ± 0.31^b^ 1.3 ± 0.22^b^	2.20 ± 0.30^a^ 1.90 ± 0.30^a^ 2.05 ± 0.21^a^	1.89 ± 0.31^a^ 2.00 ± 0.30^a^ 1.94 ± 0.22^a^	1.84 ± 0.18 1.71 ± 0.17	0.585	0.050	0.74

Means within the same row with different superscripts are significantly different (*p* ≤ 0.05)

*LSM ± SE*  Least squares means ± standard error

*Overall*: Mean value across all dietary treatments, *SE*: Standard error of the mean

The does fed *Panicum*, or Cassava during the autumn breeding season had the highest litter size (2.20 and 1.88, respectively), while those fed Berseem hay (BH) had the lowest (1.44). In addition, the higher prolificacy (litter size) was noted in does that give *Panicum* or Cassava during the spring season (1.90 and 2.00, respectively) when compared with the BH group (1.22). Furthermore, the overall means differed significantly among the experimental groups. The does fed *Panicum* or Cassava throughout the breeding seasons and gestation period depicted the highest litter size (2.05 and 1.94, respectively), whereas the lowest prolificacy was obtained by the BH group (1.33) (Table 2).

**Fig. 1:**
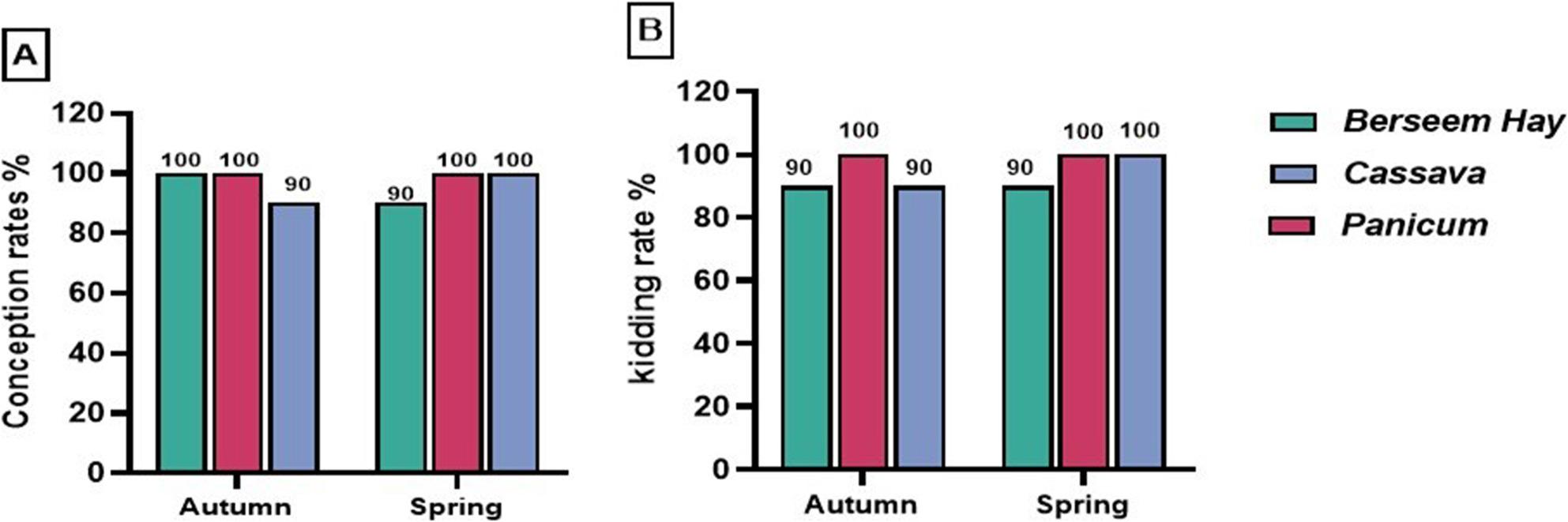
Conception (**A**) and kidding rates (**B**) in Barki does fed different types of forages during the breeding season

**Fig. 2:**
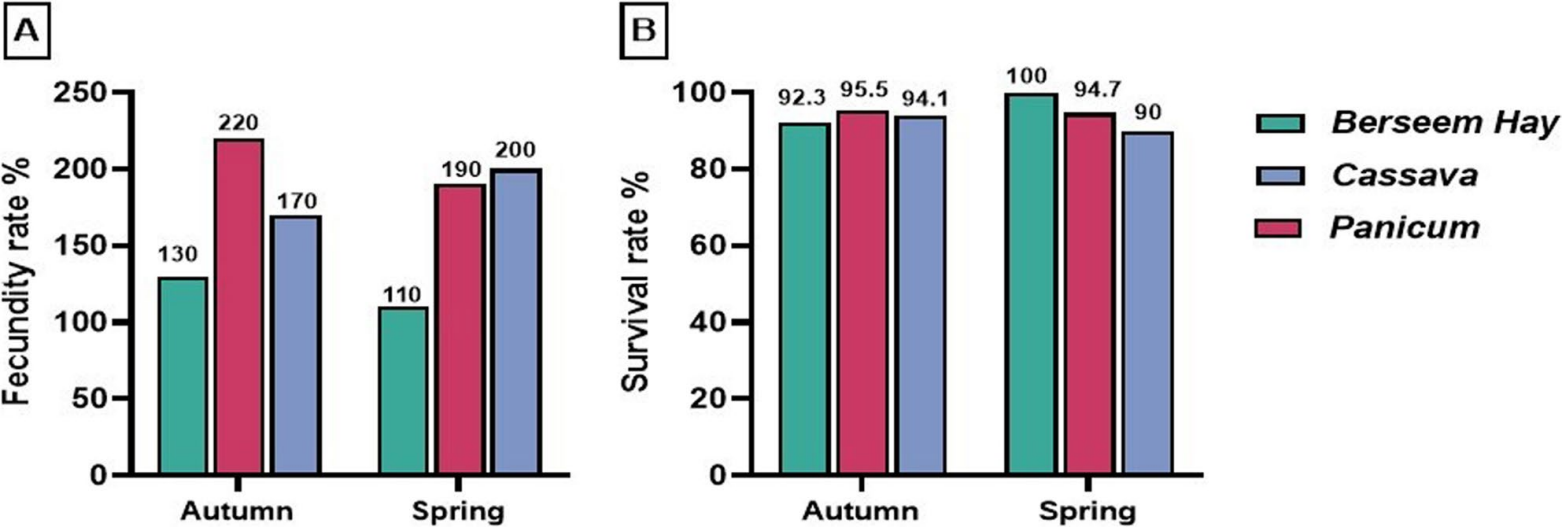
Fecundity percentages (**A**) in Barki does fed different types of forages during the breeding season, and survival rates (**B**) in kids during the suckling period

Regarding the conception rate, fed *Panicum* and Berseem hay had greater percentages (100%) than does fed Cassava (90%) in the autumn season. However, the higher conception rate (100%) during the spring season was achieved by the *Panicum* and Cassava groups when compared with the BH group (90%) (Fig. 1A). In terms of kidding rate, does fed *Panicum* in the fall breeding season had the highest value (100%) compared to their counterparts fed cassava or Berseem hay (90% for both). On the other hand, the group supplemented by cassava and *Panicum* in springtime exhibited the highest percentage compared to the BH group (100% vs. 90%) (Fig. 1B). Concerning the fecundity percentage, the highest value was obtained by does fed *Panicum in autumn*, representing 220% vs. 170% for Cassava, and 130% for does fed Berseem hay. Moreover, feeding Cassava *and Panicum* during spring was associated with greater fecundity percentage (200% and 190%, respectively), while the lowest percentage was recorded in the control group (110%) (Fig. 2A).

Concerning the effect of feeding various types of forages during the breeding season on the kids’ survival rate, the kid survivability percentage in does fed *Panicum* or Cassava throughout the fall months recorded greater values (95.5 and 94.1% for *Panicum* and Cassava, respectively) compared with the BH group (92.3%). In contrast, does fed Berseem hay in the springtime had a greater percentage of their kids that survived (100%) than those of other experimental groups (94.7% for *Panicum* and 90% for Cassava) (Fig. 2B).

### Blood parameters

#### Blood hematology

Feeding different types of forages during the autumn and spring seasons revealed significant differences in the red blood cell content among the experimental groups. The RBC’s content was higher (*P* < 0.05) in the Cassava group in both breeding seasons, followed by the BH group, while the lowest value was obtained by does fed *Panicum.* Moreover, does fed Cassava recorded the highest overall means, while does in the *Panicum* group obtained the lowest value (Table 3).

**Table 3:** The hematological values (LSM ± SE) of pregnant Barki does fed different types of forages during the breeding season

Item	Season	Experimental groups			Overall	*P*-value		
		Berseem hay	Panicum	Cassava		Season	Group	Interaction
RBCs (10^6^ /mm^3^)	Autumn Spring Overall	7.03 ± 0.14^b^ 6.90 ± 0.14^b^ 6.97 ± 0.09^b^	5.99 ± 0.14^c^ 6.04 ± 0.14^c^ 6.02 ± 0.09^c^	8.13 ± 0.14^a^ 8.02 ± 0.14^a^ 8.08 ± 0.09^a^	7.05 ± 0.08 6.99 ± 0.08	0.5888	< 0.0001	0.8200
WBCs (10^3^ /mm^3^)	Autumn Spring Overall	8.06 ± 0.38^b^ 7.75 ± 0.38^b^ 7.91 ± 0.27^b^	10.6 ± 0.38^a^ 9.07 ± 0.38^a^ 9.81 ± 0.27^a^	9.51 ± 0.38^a^ 8.91 ± 0.38^a^ 9.21 ± 0.27^a^	9.38 ± 0.22^*^ 8.58 ± 0.22	0.0102	< 0.0001	0.2701
Hb (g/dL)	Autumn Spring Overall	10.2 ± 0.21^b^ 9.47 ± 0.21^b^ 9.83 ± 0.15^b^	8.54 ± 0.21^c^ 8.43 ± 0.21^c^ 8.49 ± 0.15^c^	11.7 ± 0.21^a^ 10.8 ± 0.21^a^ 11.3 ± 0.15^a^	10.2 ± 0.12^*^ 9.56 ± 0.12	0.0006	< 0.0001	0.1259
HCT (%)	Autumn Spring Overall	26.6 ± 0.26^b^ 25.7 ± 0.26^b^ 26.1 ± 0.18^b^	23.8 ± 0.26^c^ 23.5 ± 0.26^c^ 23.7 ± 0.18^c^	29.2 ± 0.26^a^ 28.3 ± 0.26^a^ 28.8 ± 0.18^a^	26.5 ± 0.15^*^ 25.8 ± 0.15	0.0012	< 0.0001	0.4820
MCV (fL)	Autumn Spring Overall	37.7 ± 0.75^b^ 37.7 ± 0.75^b^ 37.7 ± 0.53^b^	40.5 ± 0.75^a^ 39.8 ± 0.75^a^ 40.1 ± 0.53^a^	36.1 ± 0.75^b^ 35.5 ± 0.75^c^ 35.8 ± 0.53^c^	38.1 ± 0.43 37.7 ± 0.43	0.4417	< 0.0001	0.8744
MCH (pg)	Autumn Spring Overall	14.6 ± 0.41 13.9 ± 0.41 14.3 ± 0.29	14.5 ± 0.41 14.3 ± 0.41 14.4 ± 0.29	14.5 ± 0.41 13.6 ± 0.41 14.0 ± 0.29	14.6 ± 0.24 13.9 ± 0.24	0.0659	0.6264	0.6845
MCHC (g/dl)	Autumn Spring Overall	38.3 ± 0.59^b^ 36.8 ± 0.59^ab^ 37.5 ± 0.41^b^	35.9 ± 0.59^c^ 35.8 ± 0.59^b^ 35.9 ± 0.41^c^	39.9 ± 0.59^a^ 38.0 ± 0.59^a^ 39.0 ± 0.41^a^	38.1 ± 0.34^*^ 36.9 ± 0.34	0.0125	< 0.0001	0.2464
Lymphocytes (%)	Autumn Spring Overall	59.7 ± 1.00^a^ 59.3 ± 1.00^a^ 59.5 ± 0.71^a^	53.3 ± 1.00^b^ 54.3 ± 1.00^b^ 53.8 ± 0.71^c^	55.1 ± 1.00^b^ 57.4 ± 1.00^a^ 56.2 ± 0.71^b^	56.0 ± 0.58 57.0 ± 0.58	0.2195	< 0.0001	0.4303
Monocytes (%)	Autumn Spring Overall	8.46 ± 0.45^a^ 8.15 ± 0.45^a^ 8.31 ± 0.32^a^	5.97 ± 0.45^b^ 4.92 ± 0.45^b^ 5.45 ± 0.32^b^	9.31 ± 0.45^a^ 8.49 ± 0.45^a^ 8.89 ± 0.32^a^	7.92 ± 0.26^*^ 7.19 ± 0.26	0.0470	< 0.0001	0.6986
Granulocytes (%)	Autumn Spring Overall	31.9 ± 1.03^c^ 32.5 ± 1.03^b^ 32.2 ± 0.73^c^	40.72 ± 1.03^a^ 40.74 ± 1.03^a^ 40.73 ± 0.73^a^	35.6 ± 1.03^b^ 34.1 ± 1.03^b^ 34.9 ± 0.73^b^	36.1 ± 0.59 35.9 ± 0.59	0.7425	< 0.0001	0.5778

Means within the same row with different superscripts are significantly different (*p* ≤ 0.05)

Mean values sharing the same superscript letter are not significantly different, whereas different letters denote a significant difference at *p* ≤ 0.05

The asterisk (*) indicates a statistically significant difference compared to the control group at *p* ≤ 0.05

*RBC’s * Red blood cells, *WBC’s * White blood cells, *Hb * Hemoglobin, *HCT * Hematocrit, *MCV * Mean corpuscular volume, *MCH * Mean corpuscular hemoglobin, *MCHC * Mean corpuscular hemoglobin concentration, *LSM ± SE * Least squares means ± standard error. *Overall*: Mean value across all dietary treatments. *SE*: Standard error of the mean

Concerning WBC’s content, a higher value was obtained in fall months compared to springtime (9.37 vs. 8.57, 10^3^/mm^3^). The does fed *Panicum* or Cassava in both breeding seasons showed the highest WBC content, while the lowest value was obtained by the BH group (Table 3).

The hemoglobin content and hematocrit percentage differed significantly between seasons, where the autumn season showing the highest values. The present study revealed an increase in the hemoglobin content and hematocrit percentage in the does fed Cassava, while the lowest values were obtained by the *Panicum* group (Table 3).

Concerning the mean corpuscular volume (MCV), *Panicum* group showed the highest values in both breeding seasons, while the does fed Cassava recorded the lowest value. The obtained results showed insignificant differences in the mean corpuscular hemoglobin (MCH) among the experimental groups and between seasons. Regarding to mean corpuscular hemoglobin concentration (MCHC) in the blood, there was an increase in MCHC content in the experimental groups during the autumn breeding season. Animals fed Cassava during both intervals represented the highest value, while the lowest value was obtained by the *Panicum* group. Moreover, the overall mean was significantly higher for the Cassava group (*P* < 0.0001), and the does fed *Panicum* recorded the lowest value (Table 3).

The present results showed a significant increase in the lymphocyte percentages in does fed Berseem hay in both breeding seasons and in those fed Cassava in spring months. On the other hand, the lowest values were obtained for does fed *Panicum* or Cassava during autumn months and those fed *Panicum* during springtime. The overall mean differed (*P* < 0.0001) among the experimental groups, where the highest value was obtained by the BH group, followed by the Cassava group, while does fed *Panicum* recorded the lowest value (Table 3).

Feeding different types of forages during the breeding season resulted in a significant increase in the monocyte percentages in the fall season compared to the spring season. The monocyte percentages increased significantly in does fed Berseem hay or Cassava in both breeding seasons, while the lowest values were obtained by the *Panicum* group. On the other hand, *Panicum* group showed a significant increase in granulocyte percentages in both seasons, and the lowest values were obtained by does in the BH group (Table 3).

#### Protein fractions and energy components

As demonstrated in Table 4, Feeding Cassava during breeding seasons showed a significant increase in protein fractions and concentrations of some energy components in the blood (total proteins, albumin, globulin, and albumin to globulin ratio). Moreover, blood glucose and ATP levels showed a significant increase in the does fed cassava during both breeding seasons. On the other hand, the lowest overall means of total protein, albumin, globulin, glucose, and ATP were obtained by the does fed *Panicum*. While the *Panicum* group showed a significant increase in the albumin to globulin ratio in both breeding seasons, while control does had higher (*P* < 0.01) globulin levels in both intervals. Feeding does various types of forages during the spring months resulted in a significant increase in blood plasma glucose level when compared with that in the autumn breeding season (48.8 vs. 44.6, mg/dl) (Table 4).

**Table 4:** Protein fractions and concentrations of some energy components in the blood plasma (LSM ± SE) of pregnant Barki does fed different types of forages during the breeding season

Item	Season	Experimental groups				Overall	*P*-value		
		Berseem hay		Panicum	Cassava		Season	Group	Interaction
Total Protein (g/dl)	Autumn Spring Overall	7.61 ± 0.16^b^ 7.99 ± 0.16^b^ 7.79 ± 0.11^b^	6.89 ± 0.16^c^ 6.78 ± 0.16^c^ 6.82 ± 0.11^c^		8.65 ± 0.16^a^ 8.87 ± 0.16^a^ 8.76 ± 0.11^a^	7.71 ± 0.09 7.88 ± 0.09	0.1664	< 0.0001	0.3251
Albumin (g/dl)	Autumn Spring Overall	3.31 ± 0.10^b^ 3.51 ± 0.10^b^ 3.41 ± 0.07^b^	3.44 ± 0.10^b^ 3.21 ± 0.10^c^ 3.32 ± 0.07^b^		4.17 ± 0.10^a^ 4.29 ± 0.10^a^ 4.23 ± 0.07^a^	3.64 ± 0.06 3.67 ± 0.06	0.7213	< 0.0001	0.0672
Globulin (g/dl)	Autumn Spring Overall	4.30 ± 0.12^a^ 4.48 ± 0.12^a^ 4.39 ± 0.09^a^	3.42 ± 0.12^b^ 3.58 ± 0.12^b^ 3.49 ± 0.09^b^		4.48 ± 0.12^a^ 4.58 ± 0.12^a^ 4.53 ± 0.09^a^	4.07 ± 0.07 4.22 ± 0.07	0.1381	< 0.0001	0.9476
A/G ratio	Autumn Spring Overall	0.784 ± 0.03^b^ 0.805 ± 0.03^b^ 0.794 ± 0.02^b^	1.01 ± 0.03^a^ 0.949 ± 0.03^a^ 0.980 ± 0.02^a^		0.945 ± 0.03^a^ 0.942 ± 0.03^a^ 0.943 ± 0.02^a^	0.91 ± 0.02 0.89 ± 0.02	0.5943	< 0.0001	0.4684
Glucose (mg/dl)	Autumn Spring Overall	42.0 ± 2.57^b^ 48.3 ± 2.57^b^ 45.1 ± 1.82^b^	30.8 ± 2.57^c^ 33.0 ± 2.57^c^ 31.9 ± 1.82^c^		61.2 ± 2.57^a^ 65.1 ± 2.57^a^ 63.2 ± 1.82^a^	44.7 ± 1.48 48.8 ± 1.48^*^	0.0504	< 0.0001	0.7181
ATP (ng/ml)	Autumn Spring Overall	9.88 ± 0.16^b^ 9.72 ± 0.16^b^ 9.81 ± 0.11^b^	9.48 ± 0.16^b^ 9.07 ± 0.16^c^ 9.27 ± 0.11^c^		11.1 ± 0.16^a^ 11.2 ± 0.16^a^ 11.2 ± 0.11^a^	10.1 ± 0.09 10.0 ± 0.09	0.3193	< 0.0001	0.2053

Means within the same row with different superscripts are significantly different (*p* ≤ 0.05)

Mean values sharing the same superscript letter are not significantly different, whereas different letters denote a significant difference at *p* ≤ 0.05

The asterisk (*) indicates a statistically significant difference compared to the control group at *p* ≤ 0.05

*Al/Gl ratio * Albumin to globulin ratio, *ATP * Adenine tri-phosphate, *LSM ± SE * Least squares means ± standard error. *Overall*: Mean value across all dietary treatments. *SE*: Standard error of the mean

#### Liver and kidney enzymes and thyroid hormones

As presented in Table 5, the overall mean values of does fed *Panicum* recorded the highest activity of ALT and AST enzymes (35.2 and 58.3, U/L, respectively) while does in the BH group obtained the lowest values (20.4 and 37.6, U/L, in order). Moreover, concentrations of liver function enzymes GGT and ALK-P were significantly higher in the *Panicum* group *(*41.1 and 82.2, U/L, respectively), while does in the Cassava and BH groups obtained the lowest levels of both enzymes. The treatment effect revealed a significant difference between seasons in levels of AST (*P* = 0.0197) and ALK-P (*P* = 0.0004). The spring season showed increased levels of AST and ALK-P enzymes (46.5 and 69.1, U/L, one-to-one) when compared with the autumn breeding season (Table 5).

**Table 5:** Concentrations of liver and kidney enzymes and thyroid hormones (LSM ± SE) of pregnant Barki does fed different types of forages during the breeding season

Item	Season	Experimental groups			Overall	*P*-value		
		Berseem hay	Panicum	Cassava		Season	Group	Interaction
Liver functions								
ALT (U/L)	Autumn Spring Overall	20.4 ± 0.80^c^ 20.5 ± 0.80^b^ 20.4 ± 0.57^c^	34.3 ± 0.80^a^ 36.1 ± 0.80^a^ 35.2 ± 0.57^a^	24.5 ± 0.80^b^ 22.7 ± 0.80^b^ 23.6 ± 0.57^b^	26.4 ± 0.46 26.5 ± 0.46	0.9442	< 0.0001	0.0898
AST (U/L)	Autumn Spring Overall	36.8 ± 1.15^c^ 38.4 ± 1.15^b^ 37.6 ± 0.81^c^	55.4 ± 1.15^a^ 61.1 ± 1.15^a^ 58.3 ± 0.81^a^	40.7 ± 1.15^b^ 40.1 ± 1.15^b^ 40.4 ± 0.81^b^	44.3 ± 0.66 46.5 ± 0.66^*^	0.0197	< 0.0001	0.0216
GGT (U/L)	Autumn Spring Overall	27.6 ± 1.26^b^ 27.2 ± 1.26^b^ 27.4 ± 0.89^b^	41.8 ± 1.26^a^ 40.5 ± 1.26^a^ 41.1 ± 0.89^a^	30.2 ± 1.26^b^ 27.8 ± 1.26^b^ 29.1 ± 0.89^b^	33.2 ± 0.72 31.8 ± 0.72	0.1775	< 0.0001	0.6911
ALP (U/L)	Autumn Spring Overall	56.4 ± 1.79^b^ 61.9 ± 1.79^b^ 59.2 ± 1.27^b^	78.9 ± 1.79^a^ 85.5 ± 1.79^a^ 82.2 ± 1.27^a^	55.9 ± 1.79^b^ 59.8 ± 1.79^b^ 57.8 ± 1.27^b^	63.8 ± 1.03 69.1 ± 1.03^*^	0.0004	< 0.0001	0.7639
Kidney functions								
Creatinine (mg/dl)	Autumn Spring Overall	1.08 ± 0.07^b^ 1.01 ± 0.07^b^ 1.05 ± 0.05^b^	1.63 ± 0.07^a^ 1.51 ± 0.07^a^ 1.57 ± 0.05^a^	1.22 ± 0.07^b^ 1.14 ± 0.07^b^ 1.18 ± 0.05^b^	1.31 ± 0.04 1.22 ± 0.04	0.1053	< 0.0001	0.9475
BUN (mmol/L)	Autumn Spring Overall	12.1 ± 0.38^c^ 12.5 ± 0.38^c^ 12.3 ± 0.27^c^	17.2 ± 0.38^a^ 16.6 ± 0.38^a^ 16.9 ± 0.27^a^	13.5 ± 0.38^b^ 13.7 ± 0.38^b^ 13.6 ± 0.27^b^	14.2 ± 0.22 14.3 ± 0.22	0.9476	< 0.0001	0.3768
Thyroid hormones								
T3 (ng/ml)	Autumn Spring Overall	75.8 ± 2.02^a^ 103.3 ± 2.02^a^ 89.5 ± 1.43^a^	63.6 ± 2.02^b^ 93.7 ± 2.02^b^ 78.7 ± 1.43^b^	58.9 ± 2.02^b^ 86.7 ± 2.02^c^ 72.8 ± 1.43^c^	66.1 ± 1.16 94.6 ± 1.16^*^	< 0.0001	< 0.0001	0.7654
T4 (ng/ml)	Autumn Spring Overall	7.11 ± 0.27^a^ 5.44 ± 0.27 6.28 ± 0.19^a^	5.21 ± 0.27^b^ 5.15 ± 0.27 5.18 ± 0.19^b^	5.29 ± 0.27^b^ 5.06 ± 0.27 5.18 ± 0.19^b^	5.87 ± 0.16^*^ 5.22 ± 0.16	0.0029	< 0.0001	0.0054

Means within the same row with different superscripts are significantly different (*p* ≤ 0.05)

Mean values sharing the same superscript letter are not significantly different, whereas different letters denote a significant difference at *p* ≤ 0.05

The asterisk (*) indicates a statistically significant difference compared to the control group at *p* ≤ 0.05.

*ALT * Alanine transaminase, *AST*  Aspartate transaminase, *GGT * Gamma glutamyl transferase, *ALP * Alkaline phosphatase, *BUN * Blood urea nitrogen, *T3 * Triiodothyronine, *T4 * Thyroxine, *LSM ± SE * Least squares means ± standard error. *Overall*: Mean value across all dietary treatments. *SE*: Standard error of the mean

Regarding kidney function, the does fed *Panicum* showed higher overall means of creatinine and blood urea nitrogen levels (1.57 mg/dl and 16.9 mmol/L, respectively) when compared with the other experimental groups (Table 5).

The treatment effect revealed significant differences between seasons in levels of thyroid hormones (T3 and T4). The activity of T3 hormone increased significantly in does fed BH, *Panicum*, or Cassava during the spring months (94.6, ng/ml), while the autumn-breeding season showed an increased level of T4 hormone (5.9, ng/ml). The does fed BH showed significantly higher levels of T3 activity in both seasons. On the other hand, the Cassava and *Panicum* groups obtained lower T3 activity in the autumn season, while the Cassava group obtained the lowest T3 in the springtime. Moreover, there were significant differences among the experimental groups in the overall mean T3 (89.5, 78.7, and 72.8 ng/ml) for BH, *Panicum*, and Cassava, respectively (Table 5).

Concerning T4 levels, does fed Berseem hay during the autumn season recorded the highest levels (7.11, ng/ml) while *Panicum and* Cassava groups obtained the lowest levels (5.22 and 5.297, ng/ml, in order). On the other hand, T4 levels differ insignificantly among the experimental groups during the spring season. The overall means of T4 hormone were significantly higher in the does fed BH, while the *Panicum and* Cassava groups recorded the lowest values (Table 5).

#### Blood immunological parameters

Concerning the immunological parameters of pregnant Barki goats that were shown in Table 6, there were significant differences among the experimental groups in the total antioxidant capacity (TAC). Does fed *Panicum* had the highest TAC levels in both seasons, while the BH group showed the lowest values. Moreover, the overall means were 0.93, 0.66, and 0.39 mM/L for *Panicum*, Cassava, and BH, respectively. Also, *Panicum* group recorded higher Interleukin-1 (IL-1) in both seasons, whereas the animals fed Berseem hay obtained the lowest values. The overall means of IL-1 were 32.2, 28.2, and 22.1 (pg/mg) for *Panicum*, Cassava, and BH groups, respectively. Concerning IL-2 and IL-6, *Panicum*, Cassava, and BH during the spring months recorded significantly higher levels compared with those fed during autumn. The overall means of IL-2 differed significantly among the experimental groups, where the highest value (49.8, pg/mg) was recorded for the BH group and the lowest value (20.036, pg/mg) was obtained by the *Panicum* group. The concentrations of IL-6 differed significantly among the experimental groups during the spring season (Table 6). Moreover, a higher level was obtained by *Panicum* than by those of Cassava and the BH groups. On the other hand, the Cassava group achieved a higher overall mean of TNF-α, while animals fed BH obtained a lower value, being 15.9 vs. 13.2 pg/ml. Concerning the IgG, the autumn season showed a higher (*P* < 0.0) level compared with the springtime. A higher overall mean of IgG was recorded by the does fed *Panicum* (3.85, g/ml) when compared with the other experimental groups, with values being 2.70 and 2.17, g/ml for Cassava and BH groups, respectively. Moreover, *Panicum* group showed significantly higher overall means of complement components 3 and 4 (C3 and C4) (3.03 and 4.29, mg/ml, respectively). The BH group obtained the lowest C3 level, and both the Cassava and BH groups recorded the lowest C4 values (Table 6).

**Table 6:** Concentrations of some blood immunological parameters (LSM ± SE) of pregnant Barki does fed different types of forages during the breeding season

Item	Season	Experimental groups				Overall	*P*-value		
		Berseem hay		Panicum	Cassava		Season	Group	Interaction
TAC (mM/L)	Autumn Spring Overall	0.373 ± 0.04^c^ 0.398 ± 0.04^c^ 0.385 ± 0.02^c^	0.961 ± 0.04^a^ 0.905 ± 0.04^a^ 0.933 ± 0.02^a^		0.631 ± 0.04^b^ 0.691 ± 0.04^b^ 0.661 ± 0.02^b^	0.655 ± 0.02 0.664 ± 0.02	0.7384	< 0.0001	0.2395
IL-1 (pg/ml)	Autumn Spring Overall	21.48 ± 1.00^c^ 22.85 ± 1.00^c^ 22.16 ± 0.71^c^	32.18 ± 1.00^a^ 32.13 ± 1.00^a^ 32.15 ± 0.71^a^		28.83 ± 1.00^b^ 27.55 ± 1.00^b^ 28.19 ± 0.71^b^	27.49 ± 0.58 27.51 ± 0.58	0.9843	< 0.0001	0.4226
IL-2 (pg/ml)	Autumn Spring Overall	46.24 ± 2.10^a^ 53.43 ± 2.10^a^ 49.83 ± 1.48^a^	18.42 ± 2.10^c^ 21.65 ± 2.10^c^ 20.04 ± 1.48^c^		25.43 ± 2.10^b^ 31.33 ± 2.10^b^ 28.38 ± 1.48^b^	30.03 ± 1.21 35.47 ± 1.21^*^	0.0017	< 0.0001	0.6294
IL-6 (pg/ml)	Autumn Spring Overall	14.55 ± 2.99 20.98 ± 2.99^b^ 17.77 ± 2.11^b^	22.12 ± 2.99 43.28 ± 2.99^a^ 32.70 ± 2.11^a^		15.79 ± 2.99 22.59 ± 2.99^b^ 19.19 ± 2.11^b^	17.48 ± 1.73 28.95 ± 1.73^*^	< 0.0001	< 0.0001	0.0209
TNF-α (pg/ml)	Autumn Spring Overall	12.53 ± 1.09 13.77 ± 1.09^b^ 13.15 ± 0.77^b^	13.64 ± 1.09 14.59 ± 1.09^b^ 14.12 ± 0.77^ab^		15.11 ± 1.09 16.70 ± 1.09^a^ 15.91 ± 0.77^a^	13.76 ± 0.63 15.02 ± 0.63	0.1567	0.0386	0.9572
IgG (mg/dl)	Autumn Spring Overall	2.84 ± 0.39^b^ 1.49 ± 0.39^b^ 2.17 ± 0.28^b^	4.84 ± 0.39^a^ 2.86 ± 0.39^a^ 3.85 ± 0.28^a^		3.28 ± 0.39^b^ 2.12 ± 0.39^ab^ 2.70 ± 0.28^b^	3.65 ± 0.23^*^ 2.16 ± 0.23	< 0.0001	0.0001	0.5592
C3 (mg/ml)	Autumn Spring Overall	0.888 ± 0.18^c^ 1.09 ± 0.18^c^ 0.987 ± 0.13^c^	3.23 ± 0.18^a^ 2.82 ± 0.18^a^ 3.03 ± 0.13^a^		2.07 ± 0.18^b^ 1.67 ± 0.18^b^ 1.87 ± 0.13^b^	2.06 ± 0.10 1.86 ± 0.10	0.1612	< 0.0001	0.1523
C4 (mg/ml)	Autumn Spring Overall	2.28 ± 0.37^b^ 2.40 ± 0.37^b^ 2.34 ± 0.26^b^	4.07 ± 0.37^a^ 4.51 ± 0.37^a^ 4.29 ± 0.26^a^		2.89 ± 0.37^b^ 2.55 ± 0.37^b^ 2.73 ± 0.26^b^	3.08 ± 0.21 3.15 ± 0.21	0.8157	< 0.0001	0.5727

Means within the same row with different superscripts are significantly different (*p* ≤ 0.05)

Mean values sharing the same superscript letter are not significantly different, whereas different letters denote a significant difference at *P* ≤ 0.05

The asterisk (*) indicates a statistically significant difference compared to the control group at *P* ≤ 0.05

*TAC * Total antioxidant capacity, *IL-1 * Interleukin-1, *IL-2 * Interleukin-2, *IL-6 * Interleukin-6, *TNF-α * Tumor necrosis factor, *IgG * Immunoglobulin G, *C3 * Complement component 3, *C4 * Complement component 4, *LSM ± SE * Least squares means ± standard error

*Overall*: Mean value across all dietary treatments. *SE*: Standard error of the mean

## Discussion

The results of this study largely supported the hypothesis that *Spanish Panicum* and cassava leaf meals could serve as suitable alternatives to Berseem hay during the breeding season, with both forages maintaining reproductive performance while eliciting distinct physiological responses in Barki does. Specifically, *Panicum* feeding enhanced reproductive outcomes, including conception and kidding rates, fecundity, and kid survival, particularly during the autumn season, indicating its strong potential to support reproductive efficiency under varying environmental conditions. In contrast, cassava leaf meal significantly improved hematological indices (RBC, Hb, PCV, MCHC, and total leukocyte count) and plasma biochemical parameters (total protein, albumin, globulin, glucose, and ATP; *P* < 0.0001), reflecting superior protein metabolism and overall physiological health. Both forages also enhanced immune and antioxidant markers, including total antioxidant capacity (TAC), immunoglobulin G (IgG), and complement proteins C3 and C4, confirming their roles in strengthening immunity and oxidative balance. Collectively, these findings indicate that while both *Panicum* and cassava can effectively substitute Berseem hay, the selection of forage can be tailored to specific production goals, *Panicum* to optimize reproductive performance and kid survival, and cassava to support metabolic health during high-demand periods such as late gestation and early lactation. Furthermore, these results highlight the practical feasibility of incorporating locally available and cost-effective alternative forages to mitigate seasonal shortages of high-quality roughages in small ruminant feeding systems.

The production of some indigenous breeds of goat in Egypt, like Barki goats, is hindered due to poor nutrient intake as a result of seasonal variation and the high cost of feed. Berseem (*Trifolium Alexandrinum*) is the traditional winter forage in the Mediterranean and Middle East regions. In Egypt, Berseem has achieved the distinction of being a base for livestock production due to its high nutritive value and easy cultivation. However, the absence of berseem during the summer season and the high cost of feed are limiting factors for livestock and goat production. This has necessitated the need to search for readily available, cheap, and friendly feed supplements to overcome forage shortages and reduce feed costs. For this reason, the primary objective of this study is to ascertain whether providing *Panicum* or cassava to Bakri goats during the breeding season could be a good alternative to berseem hay and their effects on reproductive performance and physiological parameters.

Reproductive performance is a key determinant for the efficiency of goat production [23]. Stresses such as malnutrition or extremes of temperature may result in either death, stillbirths, or even abortions of pregnancies, where kidding success is an important contributor to goat production [24]. This may, in turn, further threaten the reproductive potential of goat herds that resulting in the shortage of female kids that can replace older and unproductive females [25]. In the current study, the inclusion of *Panicum* and cassava in the diet of Barki goats resulted in improvements in the majority of reproductive indicators, where the litter size of female goats fed on *Panicum and* cassava was significantly larger than the BH group during both seasons. Larger litter size in *Panicum* and cassava groups may be related to an increased number of ova, which is a major component of the litter size [26]. Our results are in correspondence with [27], who reported that incorporation of *Panicum maximum* in the diet of guinea pigs increased in litter size and viability. Also [28], detected higher litter size at birth of lamb from WAD goats fed on Cassava *Manihot esculenta* and *Acacia neloitca* groups than in *Atriplex halimus* and control. Does fed *on Panicum* and cassava exhibited a significantly higher conception and kidding rate (100%) than those fed on Bersem hay (90%) during spring. A similar trend has been spotted by [29], who verified the reproductive traits improvement as cassava hay concentration increased by 25% as a replacement of berseem hay in Barki ewes. The kid survivability percentage in does fed *Panicum* or cassava throughout the fall months recorded the greatest values compared with the BH group.

Regarding fecundity (%), the highest value was obtained by does fed *Panicum* in autumn. Moreover, feeding does Cassava *and Panicum* during spring was associated with a greater fecundity percentage. Short periods of improved nutrient supply before and during mating have been known to affect ovulation rate along with the increased size and number of follicles [30], reduce follicular atresia, affect ovarian sensitivity to gonadotropins [31], and alter plasma gonadotropin concentration [32]. Additionally, The rise in conception rate and other reproductive performance parameters induced by *Panicum* and cassava confirm that female goats in our study receive the adequate requirements of nutrients, especially tannins-rich plants in ewe’s diets, which improve the live body weight, body condition, and protein absorption from the small intestine [33], as well, energy during pregnancy period that has a beneficial effect on goats’ ability to complete pregnancy and kid delivery that aligned with our findings. Furthermore, as will be covered in more detail later, this improvement might also be connected to improvements in immunity, antioxidant status, and general health in the *Panicum* and cassava *groups.* This observation is consistent with the report of [34], who observed that the inclusion of 30% cassava leaf meal in the diet of sows during pregnancy and lactation has no detrimental effects on reproduction. As well [35] and [36], found that feeding on different forage types as an alternative to BH during late pregnancy doesn’t have any adverse effects on the reproductive performance of ewes. On the contrary [37, 38], observed that adding 10–15% of ensiled cassava leaves to the diet of pregnant sows has no significant differences for most reproductive parameters. This discrepancy might have resulted from comparisons with other plants and variations in the proportion of *Panicum* or cassava in the diet.

Concerning gestation length, our results demonstrate that there were no significant differences among the *Panicum*, cassava, and Berseem hay groups in gestation length during both seasons. In the same line [39], noticed that all does fed cassava peel meal (CPM) and cassava leaf meal (CLM) (25, 50, and 75%) in substitution of wheat offal and palm kernel cake had similar gestation duration. The range of gestation length in this study was very close and consistent with the ranges reported by [40–42]. Season had no significant effect on reproductive parameters, that concurrent with [43], who revealed that there were no significant effects of breeding season of hair sheep on conception, lambing, and abortion rates. The improvements in reproductive performance observed with *Panicum* supplementation can be attributed to its balanced protein and energy content, which likely supported ovarian follicle development, ovulation, and early embryonic survival [44]. Adequate dietary protein has been shown to enhance reproductive efficiency in small ruminants by promoting gonadotropin release and luteal function [45]. Sheep and goats are more thermotolerant than cattle; however, they remain vulnerable to the direct and indirect effects of climate change. Indeed, climatic conditions can reveal negative impacts on the reproductive performances of livestock [46]. Heat stress is associated with a decrease in oocyte quality and a disturbance of the uterine environment. However, in the current study, there was no significant effect of season on reproductive performance. Our findings are consistent with [43], who revealed that there were no significant effects of breeding season on conception, lambing, and abortion rates of hair sheep. However, he detected that the wet season had higher insignificant prolificacy, mortality rates, and lambs born to ewes serviced. Conversely [47], reported a significantly higher conception rate of goats inseminated with average maximum ambient temperatures ≥ 34 °C than goats inseminated when the average maximum was lower than 34 °C for 3 days before breeding, revealing that goats mated during the spring had higher reproductive performance than goats mated during other seasons. On the other hand [48], found that sustained exposure to high temperatures (≥ 32 °C) during pregnancy decreased lamb survival under field conditions. As well [49], found an increase in fecundity and prolificacy, especially in the autumn season, in Assaf and Awassi breeds.

In general, blood biochemical indexes can be employed as markers of animal health since they show whether tissue and organ functioning, digestion and metabolism of nutrients, and other physiological processes are normal [50]. The current study declares the favorable effects of cassava and *Panicum* on hematological parameters during autumn and spring, where the cassava group showed a significant rise in total erythrocyte count, Hb, PCV, and MCHC values as compared to the BH group. However, the lowest value of Hb was recorded in the *Panicum* group, but the Hb concentration in this group (8.55) g/dl still falls within the normal values of 8–15 g/dl indicated by [51] and 7–15 g/dl by [52]. These values point to the absence of microcytic hypochromic anemia occasioned by iron deficiency and improper utilization for the formation of hemoglobin [53]. As well, these results indicate that cassava could enhance erythropoiesis and that *Panicum* supplementation has no negative effects on hematological parameters. Our results are in correspondence with those of [54], who noticed a rise in erythrocyte count in goats fed cassava peels and cassava leaves [55], also observed a significant effect of cassava pulp-based diets at 15 and 30% for 9 weeks on RBC, Hb, and PCV in West African dwarf (WAD) sheep. Similarly [56], recorded increased RBC, Hb, and WBCs in sheep fed a 50% CPM diet. Also, several studies didn’t notice any negative effects of cassava on hematological parameters [57] [58]. found that cassava at 30 and 60% as a replacement for brewer’s dried grains (BDG) in sheep and goats showed no significant difference in RBC, PCV, or Hb when compared to goats fed the control diet. Moreover, the study of [59] found similar values of PCV and RBC count in goats fed 15% CLM than *Gliricidia* and *Leucaena* leaf, suggesting that CLM might be rich in quality proteins required for normal blood formation. Concerning *Panicum*, similarly [60], reported that *Panicum maximum* and *Newbouldia laevis* fed to WAD goat kids between 9 and 15 months old for a 90-day had no adverse effect on the blood profile.

WBCs are essential for the immune system, particularly lymphocytes and monocytes, which are precursors of macrophages and are important for cell-mediated and humoral immunity responses [61]. The results from the current study revealed a significant rise in total leukocyte count in the cassava *and Panicum* groups when compared to the control diet group. Moreover, monocyte count showed a significant increase in cassava and Berseem hay goats as compared to *Panicum* ones. The values obtained for lymphocytes in this study fell within the broad range of 47–82% reported by [62] and [63]. These values are suggestive of a well-developed immune system [62]. The rise in lymphocyte (%) for the cassava group in spring and monocyte in both seasons (autumn and spring), with no significant differences with the Berseem hay group, might be linked to an increase in the activity of the bone marrow as well as the physiological stress of pregnancy. Our findings are supported by the study of [64], who found improvements in leukocyte count, lymphocytes, and neutrophils in WAD goats fed CPM and CPLM meals at 10, 20, and 30%. Additionally, the increased leukocyte is in line with the reports of [65, 66] in Sahel does, and [67] in WAD goats, all of whom reported a significant increase in the total leukocyte counts at the last trimester of gestation. On the other hand [54, 64, 68], reported a decline in leukocyte count and attributed that to cassava variety and the presence of Hydrogen cyanide (HCN), which is reported to limit the use of cassava in livestock feed. An investigation by [69] revealed similar blood constituents in WAD sheep fed CPM, and these blood values fell within the normal values reported by [70]. On the same line, several studies reported that *Panicum* has no negative effect on leukocyte count [60].

Additionally, progressive changes in climate conditions are likely to lead to relatively wide ranges in hematological values in sheep and goats in different geographic areas [71, 72]. In the current study, seasonal variation affects only some hematological parameters, including PCV, Hb, MCHC, WBCs, and monocytes. Their values were significantly higher in autumn than in spring. This could be due to favorable conditions during autumn. On the same line [73], reported that the highest number of WBCs was generally observed in summer and autumn and the lowest in winter and spring in Holstein dairy cows. Also [74], noticed that monocytes were significantly higher during the dry than the rainy season. On the other hand, WBCs values were not affected by season, neither in Barki does nor their kids, where the percentages of HCT or PCV were higher in autumn (27.99%) and spring (28.58%) compared to winter (26.52%) and summer (26. 75%) [73]. The erythrocyte count, MCV, MCH, and lymphocytes in this study didn’t show any significant differences during, autumn and spring seasons. Corresponding to our results [75], reported non-significant variations in MCV and MCH during different seasons in cows. Similarly [76, 77], , and [78] reported no significant seasonal variations in all hematological parameters., Contradicted to our results, the hematological profiles of Andaman local goats differed significantly between rainy and dry seasons. Where RBCs count was significantly higher in rainy than in dry seasons, whereas MCV and MCH were significantly higher during dry than rainy seasons [79].

Regarding protein fractions and concentrations of some energy components in pregnant Barki does, the current findings demonstrated that the cassava group exhibited significant rises in plasma levels of total proteins, albumin, globulin, glucose, and ATP during both seasons (spring and autumn) when compared to their counterparts of control or *Panicum* groups. The higher concentrations of blood proteins reflect a better quality of protein contained in Cassava does although of protein percentage in the cassava crop is lower than in *Panicum.* With no doubt, this reflects the positive effect of Cassava on liver function, whereas albumin is directly synthesized in the liver, and its amount in the blood is correlated with the function of the liver [80] [81]. mentioned that Cassava foliage meal can be an alternate unconventional protein source for replacing soybean meal in growing rabbits’ diets. This may be attributed to that cassava leaf meal contains high protein content of (16.6 to 39.9%) [82]. Similar results were detected by [83], who suggested that elevated levels of blood proteins in goats fed a diet 60% CLM were replaced by brewer’s dried grains (BDG) compared to the control, which is an indication of high protein quality in a diet having 60% CLM. On the other hand [84], found that replacing clover hay in the diet of Baladi lactating goats with *Panicum* (25 and 50%) induced significant increases in total proteins and albumin. Although in the current study *Panicum* group showed lower protein and albumin values compared to the cassava and berseem hay groups.

Globulin values in the present study for cassava and Berseem hay groups are higher than the reference range of globulin (2.7–4.1 g/dl) reported by [57] for healthy goats. While does fed on *Panicum* were within the normal range. This is an indication of the proper functioning of the liver and high immunity response of pregnant Bakri does after supplementation with Cassava. Also, *Panicum* didn’t have negative effects on globulin levels. Our findings run in parallel with the study of [85], who mentioned that serum globulin level increased significantly in West African dwarf (WAD) goat after being supplemented with 60% Cassava root sievate meal. As well [84], reported a significant increase in serum globulin levels of Baladi lactating goats compared to the clover hay group and increased with increasing *Panicum* concentration (25 and 50%).

Serum blood glucose level is one of the commonest blood metabolites used to evaluate the energy status of animals [83]. Our findings revealed increased plasma levels of glucose and ATP in the cassava group in the current study, which may be attributed to that cassava is the highest source of carbohydrates after sugarcane [86]. On the same line [57], observed a high serum glucose level in goats fed CLM compared to those fed a diet without CLM. Additionally, several studies have documented the energy density and crude protein of CLM [58, 87].

Monitoring liver and kidney functions after the incorporation of *Panicum and* cassava *in* the ration of goats is an essential step to distinguish any possible side effects. ALT is an enzyme found in the highest amount in the liver and is therefore used to detect liver injury [88]. As well, AST activity is most useful in hepatic and muscular injuries. The ALP is a key enzyme associated with the metabolic activities of animals [89]. In the current study, it is worth paying attention to the fact that the cassava group maintained normal liver and kidney function at a level closer to that of the BH group, while the *Panicum* group had the highest levels of AST, ALT, GGT, ALK-P, creatinine, and BUN in plasma. On the same line [10], reported that ALT and AST activity declined as greater proportions of cassava foliage were added to the diet. The *Panicum* group in this study exhibited a significant increase in AST and ALT levels compared to the cassava and berseem groups. The activity of ALT studied was influenced by *Panicum* supplementation; however, it was within the reported reference range of 15.3–52.3 (µ/l) for goats. On the contrary [84], reported a significant decline in levels of AST and ALT in the serum of Baladi lactating goats fed *Panicum* compared to clover hay. However, this result is inconsistent with previous studies [90, 91] where ALT and AST levels did not vary among goats fed different forages. These differences may be attributed to the types and percentage of roughage used and the tolerance of the animals, and further research is needed.

High levels of urea in the serum indicate a lowered utilization of protein, poor-quality protein, or excess protein catabolism associated with protein deficiency (9,11, 14, and 16). In the current study, the *Panicum* group exhibited the highest level of plasma BUN. This suggests that meals containing berseem and cassava had superior protein quality and were more easily digested than diets with *Panicum*. Corresponding to our results [68], reported that female goats fed cassava peels exhibited reduced levels of serum urea compared to the control. Conversely [84], observed a noteworthy decrease in blood urea levels in Baladi lactating goats given *Panicum* at concentrations of 25 and 50% in contrast to clover hay. Creatinine level is directly correlated with muscle mass and kidney function [92]. Unfortunately, the mean values of creatinine level in the *Panicum* group (1.634 and 1.514, mg/dl, in autumn and spring seasons, respectively), were higher than the normal physiological range, where does in cassava and Berseem hay groups were within the normal range of 0.7–1.5 mg/dl, for apparently healthy goats [93]. Similarly, serum creatinine levels reported in goats fed CLM at 20, 40, and 60% as a replacement for BDG fell within the standard range. Also [10], detected that feeding goats on 50% cassava foliage for 70 days resulted in reducing levels of creatinine. However [84], reported a significant decline in creatinine after the addition of *Panicum* to the diet of goats. In general, the within-normal physiological range recorded in this study generally provided a clear indication of the absence of dysfunction or injury in the hepatic and renal tissues, except for a few parameters in the *Panicum* group. The enhancement of hematological and plasma biochemical parameters with cassava leaf meal was likely driven by its high crude protein, essential amino acids, and micronutrient content, which support erythropoiesis, plasma protein synthesis, and glucose metabolism [94]. Previous studies have reported similar improvements in blood profiles and metabolic indicators in small ruminants fed protein-rich forages or cassava by-products [95–97].

In this context, season differences affect glucose, which was significantly higher in spring than in autumn. However, season did not significantly affect plasma levels of total proteins, albumin, globulin, and ATP. The higher glucose levels in spring than autumn might be explained by the fact that springtime temperatures are more stressful than fall temperatures [89], which reduces the consumption of glucose. Conversely, in a previous study, assessment of energy metabolites revealed that plasma glucose significantly decreased during the summer [98]. showed declined levels of glucose and cholesterol during winter. Corresponding to our results [99], reported a non-significant effect of season variation on total protein levels in goats and sheep. However [98] and [100], revealed a significant drop in the total protein content and albumin during winter. Liver function also showed a slightly significant effect of season, where AST and ALP levels were significantly higher in spring than in autumn. However, season did not significantly affect ALT and GGT plasma levels. Corresponding to our results, AST and ALP were significantly higher during summer than winter. As well [101] and [100], reported a higher level of ALT in summer as compared to winter. *In this study*, there was no season effect on renal function since BUN and creatinine were not substantially different in either season. Contrary to the present findings, previous studies by [102] and [103] have reported significantly higher BUN during the summer. However, this difference could be attributed to our study comparing seasonal variation in autumn and spring.

Total antioxidant activity is the cumulative action of all antioxidants present in serum and body fluids, effectively referring to the dynamic equilibrium between pro-oxidant and antioxidant agents [104]. Moreover, complement is a central component of the innate immune system, which is involved in host defense against diseased agents [105]. Feeding pregnant Barki does on *Panicum* and cassava in the present study brought significant elevations in plasma levels of TAC, IgG, and C3 when compared to BH. Also, *Panicum* induced significantly higher levels of C4 than cassava and BH. However, plasma levels of TNF-α and IL-6 didn’t show any significant differences during the autumn season, but the cassava group exhibited a slight, significant rise in TNF-α during spring. This reflects the positive effect of *Panicum* and cassava on the antioxidant status and immune system of Barki goats and clarifies the improvement in reproductive performance, as it is well established that reactive oxygen species exert a biphasic effect during pregnancy, and parturition at adequate levels is fundamental for many physiological pathways to occur, such as embryo implantation [106]. The presence of many bioactive molecules in cassava foliage, such as phenols and flavonoids [107] and phenolic chemicals and gallic acid [108] in *Panicum*, may account for this impact on antioxidant status. Additionally, condensed tannins found in cassava and *Panicum* have been shown to increase the production of immunoglobulins by preventing the rumen’s breakdown of dietary protein and sulfur amino acids. This increases the absorption of glutamine, methionine, cystine, and arginine in sheep [109]. These amino acids regulate activation of T and B lymphocytes and lymphocyte functions, thus enhancing the production of immunoglobulins [109, 110]. Moreover, it was concluded in other studies that condensed tannin can directly increase the gamma-delta (γδ) T lymphocyte numbers, which support B cells, to induce a substantial increase in the production of IgG. As well [10], observed that replacement of king grass by cassava foliage silage with different concentrations (25, 50, 75, and 100%) in the diet of Hainan black goats induced rises in antioxidant status and immunity parameters, including C3, C4, IgA, IgG, and IgM, and pointed out that the 50% cassava foliage group showed the highest oxidative capacity. *Panicum* was shown to have an antioxidant impact on lactating Baladi goats, as evidenced by studies by [60, 84], who observed a significant rise in GPx levels and a decrease in MDA when compared to clover hay. As well [108], found that *Panicum* maximum has the highest capacity to scavenge free radicals when compared to *Amaranthus hybridus* and *Brachiaria brizantha*, confirming the *Panicum* antioxidant properties. The observed increases in immune and antioxidant markers (TAC, IgG, C3, and C4) in does fed both *Panicum* and cassava were mechanistically linked to the presence of bioactive compounds such as vitamins, carotenoids, and polyphenols [111, 112]. These compounds enhance immunoglobulin production, complement activation, and free radical scavenging, contributing to improved oxidative balance and disease resistance [10].

Various experiments conducted in small ruminants have also demonstrated compromised or enhanced immune cell activity during summer [113]. Lower expressions of the cytokine genes interleukin 18 (IL-18), tumor necrosis factor-α (TNF-α), interferon-β, and interferon-γ have been reported [113, 114]. In the current study, among different factors related to antioxidant, anti-inflammatory, and immunity, the breeding season affected only IgG, IL-2, and IL-6, where IgG levels were significantly higher in autumn than spring, and IL-2 and IL-6 levels showed the opposite trend. This indicates enhanced immune cell activity during autumn compared to spring.

Overall, the present study revealed distinct advantages of *Panicum* and cassava leaf meals, indicating that their selection can be tailored to specific production goals. Panicum supplementation significantly enhanced reproductive outcomes, including conception and kidding rates, fecundity, and kid survival, particularly during the autumn breeding season. These effects suggest that Panicum is most suitable when the production goal is to maximize reproductive efficiency and offspring survival. In contrast, cassava leaf meal feeding markedly improved hematological indices (RBC, Hb, PCV, MCHC, total leukocyte count) and plasma biochemical parameters (total protein, albumin, globulin, glucose, and ATP), reflecting enhanced protein metabolism and overall physiological health. Such improvements indicate that cassava is especially advantageous during late gestation and early lactation, when does has elevated metabolic and nutrient demands. Both forages also improved immune competence and antioxidant capacity, as evidenced by higher TAC, IgG, and complement proteins C3 and C4. However, the distinct physiological profiles of each forage suggest that Panicum and cassava can be strategically incorporated into feeding programs depending on the desired production outcomes and seasonal considerations.

The findings of this study provide actionable guidance for incorporating *Spanish Panicum* and cassava leaf meals into feeding programs for Barki does. That’s why farmers should consider seasonal forage availability, ensure balanced rations, and monitor animal health, such as liver enzyme activity, when *Panicum* is included over long periods. Both forages are locally available and cost-effective alternatives to Berseem hay, which can help alleviate seasonal feed shortages.

## Conclusion

This study demonstrated that replacing Berseem hay with *Spanish Panicum* or cassava leaf meals during the spring and autumn breeding seasons improved the reproductive performance, physiological status, and immune responses of Barki does, with distinct benefits for each forage. Panicum feeding enhanced conception and kidding rates, fecundity, and kid survival, particularly in the autumn season, while cassava leaf meal improved hematological indices and plasma biochemical parameters, reflecting better protein metabolism and overall physiological health. Both forages also increased antioxidant and immune markers, indicating strengthened disease resistance. The findings suggested that the choice of forage could be tailored to specific production goals: Panicum for reproductive efficiency and kid survival, and cassava for metabolic support during periods of high nutrient demand. Both forages were locally available, cost-effective alternatives to Berseem hay, capable of mitigating seasonal forage shortages. Although the study was limited to two breeding seasons and short-term assessment, it provided the first comparative evaluation of *Panicum* and cassava in Barki does. Further research is warranted to determine optimal inclusion levels, explore combined or sequential feeding strategies, and assess long-term reproductive, growth, and economic outcomes to establish sustainable feeding models for small ruminants in Egyptian production systems.

## Data Availability

The datasets used and/or analyzed during the current study are available from the corresponding author upon reasonable request.

## Abbreviations

BH: Berseem Hay
CMF: Concentrate mixture feed
PUFA: Polyunsaturated fatty acids
RBC: Red blood cell count
PCV: Packed cell volume
Hb: Hemoglobin
HCT: Hematocrit
MCV: Mean corpuscular volume
MCH: Mean corpuscular hemoglobin
MCHC: Mean corpuscular hemoglobin concentration
WBC: White blood cell count
A/G: Albumin/Globulin ratio
ALP: Alkaline phosphatase
ALT: Alanine aminotransferase
AST: Aspartate aminotransferase
GGT: Gamma-glutamyl transferase
ATP: Adenosine triphosphate
BUN: Blood urea nitrogen
CAT: Catalase
C3: Complement component 3
C4: Complement component 4
IgG: Immunoglobulin G
IL-1: Interleukin-1
IL-2: Interleukin-2
IL-6: Interleukin-6
MDA: Malondialdehyde
TAC: Total antioxidant capacity
T3: Triiodothyronine
T4: Thyroxine
TNF-α: Tumor necrosis factor-alpha
